# *δ*-Cut Decision-Theoretic Rough Set Approach: Model and Attribute Reductions

**DOI:** 10.1155/2014/382439

**Published:** 2014-07-22

**Authors:** Hengrong Ju, Huili Dou, Yong Qi, Hualong Yu, Dongjun Yu, Jingyu Yang

**Affiliations:** ^1^School of Computer Science and Engineering, Jiangsu University of Science and Technology, Zhenjiang, Jiangsu 212003, China; ^2^Key Laboratory of Intelligent Perception and Systems for High-Dimensional Information, Nanjing University of Science and Technology, Ministry of Education, Nanjing, Jiangsu 210094, China; ^3^School of Economics and Management, Nanjing University of Science and Technology, Nanjing, Jiangsu 210094, China; ^4^School of Computer Science and Engineering, Nanjing University of Science and Technology, Nanjing, Jiangsu 210093, China

## Abstract

Decision-theoretic rough set is a quite useful rough set by introducing the decision cost into probabilistic approximations of the target. However, Yao's decision-theoretic rough set is based on the classical indiscernibility relation; such a relation may be too strict in many applications. To solve this problem, a *δ*-cut decision-theoretic rough set is proposed, which is based on the *δ*-cut quantitative indiscernibility relation. Furthermore, with respect to criterions of decision-monotonicity and cost decreasing, two different algorithms are designed to compute reducts, respectively. The comparisons between these two algorithms show us the following: (1) with respect to the original data set, the reducts based on decision-monotonicity criterion can generate more rules supported by the lower approximation region and less rules supported by the boundary region, and it follows that the uncertainty which comes from boundary region can be decreased; (2) with respect to the reducts based on decision-monotonicity criterion, the reducts based on cost minimum criterion can obtain the lowest decision costs and the largest approximation qualities. This study suggests potential application areas and new research trends concerning rough set theory.

## 1. Introduction

Decision-theoretic rough set (DTRS) was proposed by Yao et al. in the early 1990s [1, 2]. Decision-theoretic rough set introduces Bayesian decision procedure and loss function into rough set. In decision-theoretic rough set, the pair of thresholds *α* and *β*, which are used to describe the tolerance of approximations, can be directly calculated by minimizing the decision costs with Bayesian theory. Following Yao's pioneer works, many theoretical and applied results related to decision-theoretic rough set have been obtained; see [3–13] for more details.

In decision-theoretic rough set, Pawlak's indiscernibility relation is a basic concept [14–19], and it is an intersection of some equivalence relations in knowledge base. It should be noticed that, in [20], Zhao et al. have made a further investigation about indiscernibility relation and proposed another two indiscernibility relations, which are referred to as weak indiscernibility and *δ*-cut quantitative indiscernibility relations, respectively. Correspondingly, Pawlak's indiscernibility relation is called the strong indiscernibility relation. By comparing such three binary relations, it is proven that the *δ*-cut quantitative indiscernibility relation is a generalization of both strong and weak indiscernibility relations. Therefore, it is interesting to construct *δ*-cut decision-theoretic rough set based on *δ*-cut quantitative indiscernibility relation. This is what will be discussed in this paper.

Furthermore, attribute reduction is one of the most fundamental and important topics in rough set theory and has drawn attention from many researchers. As far as attribute reduction in decision-theoretic rough set, the properties of nonmonotonicity and decision cost should be concerned. (1) On the one hand, as we all know, in Pawlak's rough set model, the positive region is monotonic with respect to the set inclusion of attributes. However, the monotonicity property of the decision regions with respect to the set inclusion of attributes does not hold in the decision-theoretic rough set model [21, 22]. To fill such a gap, Yao and Zhao proposed the definition of decision-monotonicity criterion based attribute reduction [23]; (2) on the other hand, decision cost is a very important notion in decision-theoretic rough set model; to deal with the minimal decision cost, Jia et al. proposed a fitness function and designed a heuristic algorithm [24].

As a generalization of decision-theoretic rough set, in our *δ*-cut decision-theoretic rough set, we conduct the attribute reductions from above two aspects. Firstly, we introduce the notion of decision-monotonicity criterion into attribute reduction and design a significance to measure attributes; secondly, to deal with the minimum decision cost problem, we regard it as an optimization problem and apply the generic algorithm to obtain a reduct with the lowest decision cost.

To facilitate our discussions, we present the basic knowledge, such as Pawlak's rough set, *δ*-cut quantitative rough set, and Yao's decision-theoretic rough set in Sections 2 and 3. In Section 4, we propose a new *δ*-cut decision-theoretic rough set and present several related properties. In Section 5, we discuss the attribute reductions by considering two criterions. The paper ends with conclusions in Section 6.

## 2. Indiscernibility Relations and Rough Sets

### 2.1. Strong Indiscernibility Relation

An information system is a pair *S* = (*U*, *AT*), in which universe *U* is a finite set of the objects; *AT* is a nonempty set of the attributes, such that for all *a* ∈ *AT*, and *V*_*a*_ is the domain of *a*. For all *x* ∈ *U*, *a*(*x*) denotes the value of *x* on *a*. Particularly, when *AT* = *C* ∪ *D* and *C*∩*D* = *∅* (*C* is the set of conditional attributes and *D* is the set of decisional attributes), the information system is also called decision system.

Each nonempty subset *A*⊆*AT* determines a strong indiscernibility relation *IND*(*A*) as follows:

(1) IND(A)={(x,y)∈U2:a(x)=a(y),∀a∈A}.

A strong indiscernibility relation with respect to *A* is denoted as *IND*(*A*). Two objects in *U* satisfy *IND*(*A*) if and only if they have the same values on all attributes in *A*; it is an equivalence relation. *IND*(*A*) partitions *U* into a family of disjoint subsets *U*/*IND*(*A*) called a quotient set of *U*:

(2) UIND(A)={[x]A:x∈U},

where [*x*]_*A*_ denotes the equivalence class determined by *x* with respect to *A*; that is,

(3) [x]A={y∈U:(x,y)∈IND(A)}.

**Definition 1 .**

Let *S* be an information system, let *A* be any subset of *AT*, and let *X* be any subset of *U*. The lower approximation of *X* denoted as ${\underline{A}}_{S}(X)$ and the upper approximation of *X* denoted as ${\bar{A}}_{S}(X)$, respectively, are defined by

(4) A_S(X)={x∈U:[x]A⊆X};A¯S(X)={x∈U:[x]A∩X≠∅}.

The pair $\left[{\underline{A}}_{S}(X),{\bar{A}}_{S}(X)\right]$ is referred to as Pawlak's rough set of *X* with respect to the set of attributes *A*.

### 2.2. Weak Indiscernibility Relation

In the definition of strong indiscernibility relation, we can observe that two objects in *U* satisfy *IND*(*A*) if and only if they have the same values on all attributes in *A*; such case may be too strict to be used in many applications. To address this issue, Zhao and Yao proposed a notion which is called weak indiscernibility relation. The semantic interpretation of weak indiscernibility relation is that two objects are considered as indistinguishable if and only if they have the same values on at least one attribute in *A*.

In an information system *S*, for any subset of *AT*, a weak indiscernibility relation can be defined as follows [20]:

(5) WIND(A)={(x,y)∈U2:a(x)=a(y),∃a∈A}.

From the description of the weak indiscernibility relation we can find that a weak indiscernibility relation *W* *IND*(*A*) with respect to *A* only requires that two objects have the same values on at least one attribute in *A*. A weak indiscernibility relation is reflexive and symmetric, but not necessarily transitive. Such a relation is known as a compatibility or a tolerance relation.

**Definition 2 .**

Let *S* be an information system; for all *A*⊆*AT*, for all *X*⊆*U*, the lower and upper approximations of *X* based on weak indiscernibility relation, denoted as ${\underline{A}}_{W}(X)$ and ${\bar{A}}_{W}(X)$, respectively, are defined by

(6) A_W(X)={x∈U:[x]AW⊆X};A¯W(X)={x∈U:[x]AW∩X≠∅},

where [*x*]_*A*_^*W*^ = {*y* ∈ *U* : (*x*, *y*) ∈ *W* *IND*(*A*)} is the set of objects, which are weak indiscernibility with *x* in terms of set of attributes *A*.

### 2.3. *δ*-Cut Quantitative Indiscernibility Relation

The strong and weak indiscernibility relations represent the two extreme cases, which include many levels of indiscernibility. With respect to a nonempty set of attributes *A*⊆*AT*, a *δ*-cut quantitative indiscernibility relation is defined as a mapping from *U* × *U* to the unit interval [0,1].

**Definition 3 (see [20]).**

Let *S* be an information system; for all *A*⊆*AT*, the *δ*-cut quantitative indiscernibility relation *ind*_*δ*_(*A*) is defined by

(7) indδ(A)={(x,y)∈U2:|{a∈A:a(x)=a(y)}||A|≥δ},

where |·| denotes the cardinality of a set.

By the definition of *δ*-cut quantitative indiscernibility relation, we can obtain the lower and upper approximations as in the following definition.

**Definition 4 .**

Let *S* be an information system; for all *A*⊆*AT*, for all *X*⊆*U*, the *δ*-cut quantitative indiscernibility based lower and upper approximations are denoted by ${\underline{A}}_{\delta }(X)$ and ${\bar{A}}_{\delta }(X)$, respectively:

(8) A_δ(X)={x∈U:[x]Aδ⊆X};A¯δ(X)={x∈U:[x]Aδ∩X≠∅},

where [*x*]_*A*_^*δ*^ = {*y* ∈ *U* : (*x*, *y*) ∈ *ind*_*δ*_(*A*)} is the set of objects, which are *δ*-cut indiscernibility with *x* in terms of set of attributes *A*.

## 3. Decision-Theoretic Rough Set

The Bayesian decision procedure deals with making a decision with minimum risk based on observed evidence. Yao and Zhou introduced a more general rough set model called a decision-theoretic rough set (DTRS) model [25–27]. In this section, we briefly introduce the original DTRS model. According to the Bayesian decision procedure, the DTRS model is composed of two states and three actions. The set of states is given by Ω = {*X*, ~*X*} indicating that an object is in *X* or not, respectively. The probabilities for these two complement states can be denoted as *P*(*X*∣[*x*]_*A*_) = |*X*∩[*x*]_*A*_|/|[*x*]_*A*_| and *P*(~*X*∣[*x*]_*A*_) = 1 − *P*(*X*∣[*x*]_*A*_). The set of actions is given by *A* = {*a*_*P*_, *a*_*B*_, *a*_*N*_}, where *a*_*P*_, *a*_*B*_, and *a*_*N*_ represent the three actions in classifying an object *x*, namely, deciding that *x* belongs to the positive region, deciding that *x* belongs to the boundary region, and deciding that*x* belongs to the negative region, respectively. The loss functions are regarding the risk or cost of actions in different states. Let *λ*_*PP*_, *λ*_*BP*_, and *λ*_*NP*_ denote the cost incurred for taking actions *a*_*P*_, *a*_*B*_, and *a*_*N*_, respectively, when an object belongs to *X*, and let *λ*_*PN*_, *λ*_*BN*_, and *λ*_*NN*_ denote the cost incurred for taking the same actions when an object belongs to ~*X*.

According to the loss functions, the expected costs associated with taking different actions for objects in [*x*]_*A*_ can be expressed as follows:

(9) RP=R(aP ∣ [x]A)=λPP·P(X ∣ [x]A)+λPN·P(~X ∣ [x]A);RB=R(aB ∣ [x]A)=λBP·P(X ∣ [x]A)+λBN·P(~X ∣ [x]A);RN=R(aN ∣ [x]A)=λNP·P(X ∣ [x]A)+λNN·P(~X ∣ [x]A).

The Bayesian decision procedure leads to the following minimum-risk decision rules:

(*P*) if *R*_*P*_ ≤ *R*_*B*_ and *R*_*P*_ ≤ *R*_*N*_, then this decides that *x* belongs to the positive region;
(*B*) if *R*_*B*_ ≤ *R*_*P*_ and *R*_*B*_ ≤ *R*_*N*_, then this decides that *x* belongs to the boundary region;
(*N*) if *R*_*N*_ ≤ *R*_*P*_ and *R*_*N*_ ≤ *R*_*B*_, then this decides that *x* belongs to the negative region.

Consider a special kind of loss functions with *λ*_*PP*_ ≤ *λ*_*BP*_ ≤ *λ*_*NP*_ and *λ*_*NN*_ ≤ *λ*_*BN*_ ≤ *λ*_*PN*_; that is to say, the loss of classifying an object *x* belonging to *X* into the positive region is no more than the loss of classifying *x* into the boundary region, and both of these losses are strictly less than the loss of classifying *x* into the negative region. The reverse order of losses is used for classifying an object not in *X*. We further assume that a loss function satisfies the following condition:

(10) (λPN−λBN)·(λNP−λBP)>(λBP−λPP)·(λBN−λNN).

Based on the above two assumptions, we have the following simplified rules:

( *P*1 ) if *P*(*X*∣[*x*]_*A*_) ≥ *α*, then this decides that *x* belongs to the positive region;
( *B*1 ) if *β* < *P*(*X*∣[*x*]_*A*_) < *α*, then this decides that *x* belongs to the boundary region;
( *N*1 ) if *P*(*X*∣[*x*]_*A*_) ≤ *β*, then this decides that *x* belongs to the negative region,

where

(11) $$\begin{array}{c} \alpha =\frac{\lambda_{PN}-\lambda_{BN}}{\left(\lambda_{PN}-\lambda_{BN}\right)+\left(\lambda_{BP}-\lambda_{PP}\right)};\\ \beta =\frac{\lambda_{BN}-\lambda_{NN}}{\left(\lambda_{BN}-\lambda_{NN}\right)+\left(\lambda_{NP}-\lambda_{BP}\right)}, \end{array}$$

with 1 ≥ *α* ≥ *β* ≥ 0.

Using these three decision rules, for all *A*⊆*AT* and for all *X*⊆*U*, we get the following probabilistic approximations:

(12) A_(α,β)(X)={x∈U:P(X ∣ [x]A)≥α};A¯(α,β)(X)={x∈U:P(X ∣ [x]A)>β}.

The pair $\left[{\underline{A}}_{\left(\alpha ,\beta \right)}(X),{\bar{A}}_{\left(\alpha ,\beta \right)}(X)\right]$ is referred to as decision-theoretic rough set of *X* with respect to the set of attributes *A*. Therefore, the positive region of *X* can be expressed as $\text{PO}{\text{S}}_{\left(\alpha ,\beta \right)}(X)={\underline{A}}_{\left(\alpha ,\beta \right)}(X)$, the boundary region of *X* is $\text{BN}{\text{D}}_{\left(\alpha ,\beta \right)}(X)={\bar{A}}_{\left(\alpha ,\beta \right)}(X)-{\underline{A}}_{\left(\alpha ,\beta \right)}(X)$, and the negative region of *X* is $\text{NE}{\text{G}}_{\left(\alpha ,\beta \right)}(X)=U-{\bar{A}}_{\left(\alpha ,\beta \right)}(X)$.

## 4. *δ*-Cut Decision-Theoretic Rough Set

As the discussion in Section 3, we can observe that the classical decision-theoretic rough set is based on the strong indiscernibility relation which is too strict since it requires that the two objects have the same values on all attributes. In this section, we introduce the concept of *δ*-cut indiscernibility relation into the decision-theoretic rough set model.

### 4.1. Definition of *δ*-Cut Decision-Theoretic Rough Set

**Definition 5 .**

Let *S* be an information system; for all *A*⊆*AT*, for all *X*⊆*U*, the decision-theoretic lower and upper approximations based on the *δ*-cut quantitative indiscernibility relation, denoted as ${\underline{A}}_{\left(\alpha ,\beta \right)}^{\delta }(X)$ and ${\bar{A}}_{\left(\alpha ,\beta \right)}^{\delta }(X)$, respectively, are defined by

(13) A_(α,β)δ(X)={x∈U:P(X ∣ [x]Aδ)≥α};A¯(α,β)δ(X)={x∈U:P(X ∣ [x]Aδ)>β}.

The pair $\left[{\underline{A}}_{\left(\alpha ,\beta \right)}^{\delta }(X),{\bar{A}}_{\left(\alpha ,\beta \right)}^{\delta }(X)\right]$ is referred to as a *δ*-cut decision-theoretic rough set of *X* with respect to the set of attributes *A*.

After obtaining the lower and upper approximations, the probabilistic positive, boundary, and negative regions are defined by

(14) POS(α,β)δ(X)=A_(α,β)δ(X);BND(α,β)δ(X)=A¯(α,β)δ(X)−A_(α,β)δ(X);NEG(α,β)δ(X)=U−POS(α,β)δ(X)∪BND(α,β)δ(X)=U−A¯(α,β)δ(X).

Let *DS* be a decision system and let *π*_*D*_ = {*D*_1_, *D*_2_,…, *D*_*t*_} be a partition of the universe *U*, which is defined by the decision attribute *D*, representing *t* classes. By the definition of quantitative decision-theoretic rough set, the lower and upper approximations of the partition can be expressed as follows:

(15) A_(α,β)δ(πD)=(A_(α,β)δ(D1),A_(α,β)δ(D2),…,A_(α,β)δ(Dt));A¯(α,β)δ(πD)=(A¯(α,β)δ(D1),A¯(α,β)δ(D2),…,A¯(α,β)δ(Dt)).

For this *t*-classes problem, it can be regarded as *t* two-class problems; following this approach, the positive region, boundary region, and negative region of all the decision classes can be expressed as follows:

(16) POS(α,β)δ(πD)=⋃i=1tPOS(α,β)δ(Di);BND(α,β)δ(πD)=⋃i=1tBND(α,β)δ(Di);NEG(α,β)δ(πD)=U−POS(α,β)δ(πD)∪BND(α,β)δ(πD).

Based on the notions of the three regions in *δ*-cut decision-theoretic rough set model, three important rules should be concerned, that is, positive rule, boundary rule, and negative rule. Similar to Yao's decision-theoretic rough set, when *α* > *β*, for all *D*_*i*_ ∈ *π*_*D*_, we can obtain the following decision rules, that is, tie-break:

(*δ*-*P*) if *P*(*D*_*i*_∣[*x*]_*A*_^*δ*^) ≥ *α*, then this decides that *x* ∈ POS_(*α*,*β*)_^*δ*^(*D*_*i*_);
(*δ*-*B*) if *β* < *P*(*D*_*i*_∣[*x*]_*A*_^*δ*^) < *α*, then this decides that *x* ∈ BND_(*α*,*β*)_^*δ*^(*D*_*i*_);
(*δ*-*N*) if *P*(*D*_*i*_∣[*x*]_*A*_^*δ*^) ≤ *β*, then this decides that *x* ∈ NEG_(*α*,*β*)_^*δ*^(*D*_*i*_).

Let *DS* be a decision system, *δ* ∈ (0,1]; for all *D*_*i*_ ∈ *π*_*D*_, the Bayesian expected costs of decision rules can be expressed as follows:

- (*δ*-*P*) cost: ∑_*D*_*i*_∈*π*_*D*__∑_*x*∈POS(*D*_*i*_)_(*λ*_*PP*_ · *P*(*D*_*i*_∣[*x*]_*A*_^*δ*^) + *λ*_*PN*_ · *P*(~*D*_*i*_∣[*x*]_*A*_^*δ*^));
- (*δ*-*N*) cost: ∑_*D*_*i*_∈*π*_*D*__∑_*x*∈NEG(*D*_*i*_)_(*λ*_*NP*_ · *P*(*D*_*i*_∣[*x*]_*A*_^*δ*^) + *λ*_*NN*_ · *P*(~*D*_*i*_∣[*x*]_*A*_^*δ*^));
- (*δ*-*B*) cost: ∑_*D*_*i*_∈*π*_*D*__∑_*x*∈BND(*D*_*i*_)_(*λ*_*BP*_ · *P*(*D*_*i*_∣[*x*]_*A*_^*δ*^) + *λ*_*BN*_ · *P*(~*D*_*i*_∣[*x*]_*A*_^*δ*^)).

Considering the special case where we assume zero cost for a correct classification, that is, *λ*_*PP*_ = *λ*_*NN*_ = 0, the decision costs of rules can be simply expressed as follows:

- (*δ*-*P*1) cost: ∑_*D*_*i*_∈*π*_*D*__∑_*x*∈POS(*D*_*i*_)_ *λ*_*PN*_ · *P*(~*D*_*i*_∣[*x*]_*A*_^*δ*^);
- (*δ*-*N*1) cost: ∑_*D*_*i*_∈*π*_*D*__∑_*x*∈NEG(*D*_*i*_)_ *λ*_*NP*_ · *P*(*D*_*i*_∣[*x*]_*A*_^*δ*^);
- (*δ*-*B*1) cost: ∑_*D*_*i*_∈*π*_*D*__∑_*x*∈BND(*D*_*i*_)_(*λ*_*BP*_ · *P*(*D*_*i*_∣[*x*]_*A*_^*δ*^) + *λ*_*BN*_ · *P*(~*D*_*i*_∣[*x*]_*A*_^*δ*^)).

For any subset of conditional attributes, the overall cost of all decision rules can be denoted as COST(*A*), such that

(17) COST(A)=COSTAPOS+COSTANEG+COSTABND=∑Di∈πD ‍∑x∈POS(Di)λPN·P(~Di ∣ [x]Aδ)+∑Di∈πD ‍∑x∈NEG(Di)λNP·P(Di ∣ [x]Aδ)+∑Di∈πD ‍∑x∈BND(Di)(λBP·P(Di ∣ [x]Aδ)        +λBN·P(~Di ∣ [x]Aδ)).

### 4.2. Related Properties

**Proposition 6 .**

Let *S* be an information system; if *λ*_*PN*_ = *λ*_*NP*_ = 1 and *λ*_*PP*_ = *λ*_*NN*_ = *λ*_*BP*_ = *λ*_*BN*_ = 0, ∀*X*⊆*U*, one has

(18) A_(α,β)δ(X)=A_δ(X);A¯(α,β)δ(X)=A¯δ(X).

**Proof**

In this proposition, we suppose that there is a unit misclassification cost if an object in *X* is classified into the negative region or if an object in ~*X* is classified into the positive region; otherwise there is no cost; that is, *λ*_*PN*_ = *λ*_*NP*_ = 1 and *λ*_*PP*_ = *λ*_*NN*_ = *λ*_*BP*_ = *λ*_*BN*_ = 0. By the computational processes of *α* and *β*, we have *α* = 1 and *β* = 0 and by the definition of *δ*-cut decision-theoretic rough set, we can observe that

(19) A_(α,β)δ(X)={x∈U:P(X ∣ [x]Aδ)≥1}={x∈U:|X∩[x]Aδ||[x]Aδ|≥1}={x∈U:[x]Aδ⊆X}=A_δ(X).

Similarly, it is not difficult to prove ${\bar{A}}_{\left(\alpha ,\beta \right)}^{\delta }(X)={\bar{A}}_{\delta }(X)$.

**Proposition 7 .**

Let *S* be an information system; for all *A*⊆*AT*, for all *X*⊆*U*, one has

(20) A_(α,β)δ(X)⊇A_δ(X);A¯(α,β)δ(X)⊆A¯δ(X).

**Proof**

For all $x\in {\underline{A}}_{\delta }(X)$ and by Definition 4, we have [*x*]_*A*_^*δ*^⊆*X*; that is to say, *P*(*X*∣[*x*]_*A*_^*δ*^) = |[*x*]_*A*_^*δ*^∩*X*|/|[*x*]_*A*_^*δ*^| = 1; since *α* ∈ (0,1], then *P*(*X*∣[*x*]_*A*_^*δ*^) ≥ *α*; by the probability, we have that $x\in {\underline{A}}_{\left(\alpha ,\beta \right)}^{\delta }(X)$ holds obviously, and it follows that ${\underline{A}}_{\left(\alpha ,\beta \right)}^{\delta }(X)⊇{\underline{A}}_{\delta }(X)$.

Similarly, it is not difficult to prove ${\bar{A}}_{\left(\alpha ,\beta \right)}^{\delta }(X)\subseteq {\bar{A}}_{\delta }(X)$.

Propositions 6 and 7 show the relationships between *δ*-cut decision-theoretic rough set and classical *δ*-cut quantitative rough set. The details are given as follows: the classical *δ*-cut quantitative indiscernibility lower approximation is included into the *δ*-cut decision-theoretic lower approximation and the *δ*-cut decision-theoretic upper approximation is included into the classical *δ*-cut quantitative indiscernibility upper approximation. Particularly, with some limitations, the *δ*-cut decision-theoretic rough set can degenerate to the classical *δ*-cut quantitative rough set. As the discussion above, we can observe that the *δ*-cut decision-theoretic rough set is a generalization of classical *δ*-cut quantitative rough set, and it can increase lower approximation and decrease upper approximation.

**Proposition 8 .**

Let *S* be an information system; if *δ* = 1, then, for all *A*⊆*AT*, for all *X*⊆*U*, one has

(21) A_(α,β)δ(X)=A_α,β(X);A¯(α,β)δ(X)=A¯α,β(X).

**Proof**

It is not difficult to prove this proposition by Definitions 3 and 5 and the definition of decision-theoretic rough set.

Proposition 8 shows the relationships between *δ*-cut decision-theoretic rough set and Yao's decision-theoretic rough set. The details are the following: if we set the value of *δ* with 1, the lower and upper approximations based on our decision-theoretic rough set are equal to those based on Yao's decision-theoretic rough set. By Proposition 8 we can observe that our decision-theoretic rough set is also a generalization of Yao's decision-theoretic rough set.

## 5. Attribute Reductions in Quantitative Decision-Theoretic Rough Set

### 5.1. Decision-Monotonicity Criterion Based Reducts

In Pawlak's rough set theory, attribute reduction is an important concept which has been addressed by many researchers all around the world. In classical rough set, the reduct is a minimal subset of attributes which is independent and has the same power as all of the attributes. The positive region, the boundary region, and the negative region are monotonic with respect to the set inclusion of attributes in classical rough set theory. However, in decision-theoretic rough set model, the monotonicity property of the decision regions with respect to the set inclusion of attributes does not hold. To solve such a problem, Yao and Zhao have proposed a decision-monotonicity criterion [23]. The decision-monotonicity criterion requires two things. Firstly, the criterion requires that by reducing attributes a positive rule is still a positive rule of the same decision. Secondly, the criterion requires that by reducing attributes a boundary rule is still a boundary rule or is upgraded to a positive rule with the same decision. Following their work, it is not difficult to introduce the decision-monotonicity criterion into our *δ*-cut decision-theoretic rough set. The detailed definition is shown in Definition 9 as follows.

**Definition 9 .**

Let *DS* = (*U*, *C* ∪ *D*) be a decision system, *δ* ∈ (0,1], and let *A* be any subset of conditional attributes; *A* is referred to as a decision-monotonicity reduct in *DS* if and only if *A* is the minimal set of conditional attributes, which preserves ${\underline{C}}_{\left(\alpha ,\beta \right)}^{\delta }(D_{i})\subseteq {\underline{A}}_{\left(\alpha ,\beta \right)}^{\delta }(D_{i})$, for each *D*_*i*_ ∈ *π*_*D*_.

Let *DS* be a decision system, *δ* ∈ (0,1], and let *A* be any subset of conditional attributes and *a*_*i*_ ∈ *A*; we define the following coefficients:

(22) DMinsig(ai,A,δ) =∑Di∈πD(A_(α,β)δ(Di)⊙A−{ai}_(α,β)δ(Di))m·t;DMoutsig(ai,A,δ) =∑Di∈πD(A_(α,β)δ(Di)⊙A∪{ai}_(α,β)δ(Di))m·t,

where *m* and *t* are the numbers of objects and decision classes, respectively, and

(23) $$\begin{array}{c} A⊙B=\{\begin{array}{cc} \left|B-A\right| & A\subseteq B,\\ -\left|A-B\right| & \text{otherwise}. \end{array} \end{array}$$

Based on these measures, we can design a heuristic algorithm to compute the decision-monotonicity reduct; the details are shown as in Algorithm 1.

### 5.2. Cost Minimum Criterion Based Reducts

Cost is one of the important features of the *δ*-cut decision-theoretic rough set. In Section 4.1 we have discussed the cost issue of our *δ*-cut decision-theoretic rough set. However, in the reduction process, from the viewpoint of cost criterion, we want to obtain a reduct with smaller or smallest cost. Similar to the decision-monotonicity criterion, it is not difficult to introduce the cost criterion into our rough set model.

**Definition 10 .**

Let *DS* = (*U*, *C* ∪ *D*) be a decision system, *δ* ∈ (0,1], and let *A* be any subset of conditional attributes; *A* is referred to as a cost reduct in *DS* if and only if *A* is the minimal set of conditional attributes, which satisfies COST(*A*) ≤ COST(*C*), and, for each set *B* ⊂ *A*, COST(*B*) > COST(*A*).

In this definition, we want to find a subset of conditional attributes so that the overall decision cost will be decreased or unchanged based on the reduct. In most situations, it is better for the decider to obtain a smaller or smallest cost in the decision procedure. We propose an optimization problem with the objective of minimizing the cost values; the minimum cost can be denoted as follows [3]:

(24) $$\begin{array}{c} \min\;\text{COST}\left(A\right). \end{array}$$

Then the optimization problem is described as finding a proper attributes set to make the whole decision cost minimum. Therefore, in the following, we will present a genetic algorithm to compute cost minimum based reducts. The details of genetic algorithm are described in Algorithm 2.

### 5.3. Experimental Analyses

In this subsection, by experimental analyses, we will illustrate the differences between Algorithms 1 and 2. All the experiments have been carried out on a personal computer with Windows 7, Intel Core 2 DuoT5800 CPU (4.00 GHz), and 4.00 GB memory. The programming language is Matlab 2012b.

We download four public data sets from UCI Repository of Machine Learning Databases, which are described in Table 1. In the experiment, 10 different groups of loss functions are randomly generated.

Tables 2, 3, 4, and 5 show the experimental results of (*P*) rules, (*B*) rules, and (*N*) rules. The number of these rules is equivalent to the number of objects in positive region, boundary region, and negative region, respectively. This is mainly because each object in positive/boundary/negative region can induce a (*P*)/(*B*)/(*N*) decision rule.

Based on these four tables, it is not difficult to draw the following conclusions.

1. With respect to the original data set, decision-monotonicity reducts can generate more (*P*) rules; this is mainly because the condition of decision-monotonicity reducts requires that, by reducing attributes, a positive rule is still a positive rule, or a boundary rule is upgraded to a positive rule. This mechanism not only keeps the original (*P*) rules unchanged, but also increases the (*P*) rules.
2. With respect to the original data set, decision-monotonicity reducts can generate less (*B*) rules; this is mainly because the second condition of decision-monotonicity reducts requires that, by reducing attributes, a boundary rule is still a boundary rule or is upgraded to a positive rule; that is to say, the number of (*B*) rules may be equal to or less than those of original data set.

**Figure 1:**
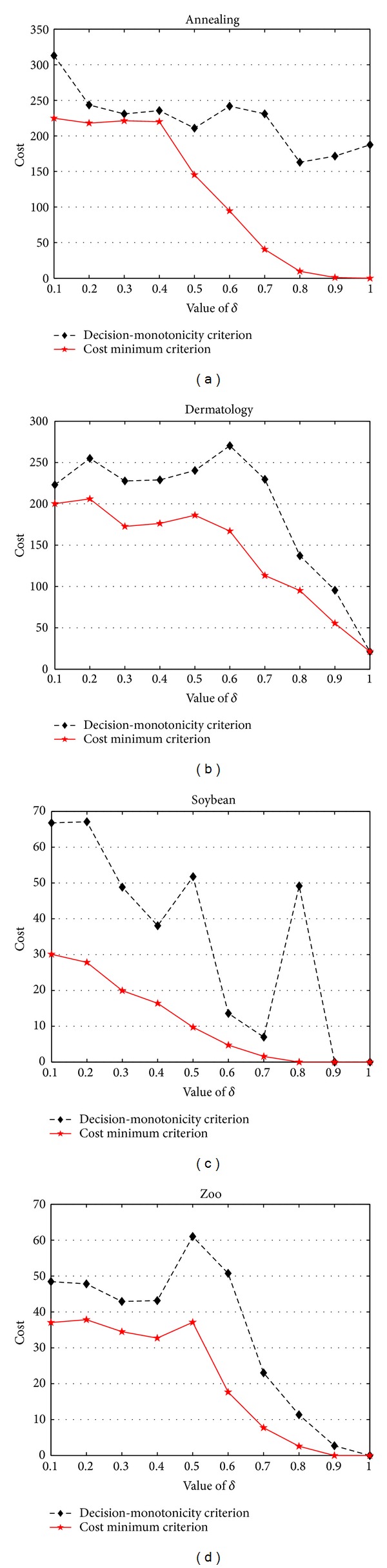
Reducts' costs comparison between decision-monotonicity and cost minimum criterions.

In order to compare the differences between decision-monotonicity criterion based reducts and cost minimum criterion based reducts, we conduct the experiments from three aspects, that is, decision costs, approximation qualities, and running times. On the one hand, Figure 1 shows the costs comparisons between these two attribute reduction algorithms; on the other hand, Tables 6, 7, 8, and 9 show the differences between decision-monotonicity criterion based reducts and cost minimum criterion based reducts in approximation qualities and running times, respectively.

In Figure 1, each subfigure is corresponding to a data set. In each subfigure, the *x*-coordinate pertains to different values of *δ*, whereas the *y*-coordinate concerns the values of costs. Through an investigation of Figure 1, it is not difficult to observe that, in all the ten used values of *δ*, the decision costs of cost minimum criterion based reducts are the same or lower than those obtained by decision-monotonicity criterion based reducts.

Tables 6 to 9 show the differences between decision-monotonicity criterion based reducts and cost minimum criterion based reducts in approximation qualities and running times, respectively. It is not difficult to note that, from the viewpoint of approximation qualities, the approximation qualities of decision-monotonicity criterion based reducts are larger than those of cost minimum criterion based reducts at times. However, in most cases, the approximation qualities of cost minimum criterion based reducts are larger than those of decision-monotonicity criterion based reducts. From the point of running times, it is easy to observe that the run times of genetic algorithm are greater than those of heuristic algorithm.

To sum up, we can draw the following conclusions.

1. From the viewpoint of decision monotonicity, our heuristic algorithm based on decision-monotonicity criterion can generate more (*P*) rules and less (*B*) rules with respect to the original data set. Such approach not only increases the certainties which are expressed by (*P*) rules and (*N*) rules, but also decreases the uncertainty coming from (*B*) rules.
2. From the viewpoint of decision costs, the generic algorithm based on cost minimum criterion can obtain the lowest decision costs and the largest approximation qualities by comparing with heuristic algorithm based on decision-monotonicity criterion. However, such approach loses the property of decision monotonicity and it wastes larger running times than heuristic algorithm.

## 6. Conclusion

In this paper, we have developed a generalized framework of decision-theoretic rough set, which is referred to as a *δ*-cut decision-theoretic rough set. Different from Yao's decision-theoretic rough set model, our model is constructed based on *δ*-cut quantitative indiscernibility relation, and it can degenerate to Yao's decision-theoretic rough set with some limitation. Based on the proposed model, we discussed the attribute reductions from two criterions; the experiments show that, on the one hand, the decision-monotonicity criterion based reducts can generate more positive rules and less boundary rules; on the other hand, the cost minimum criterion based reducts can obtain the lowest decision costs with high approximation qualities.

The present study is the first step towards *δ*-cut decision-theoretic rough set. The following are challenges for further research.

1. *δ*-cut decision-theoretic rough set approach to complicated data type, such as interval-valued data, is one of the challenges; incomplete data may be an interesting topic.
2. The threshold learning of *δ* in this paper is also a serious challenge.

## Figures and Tables

**Algorithm 1:**
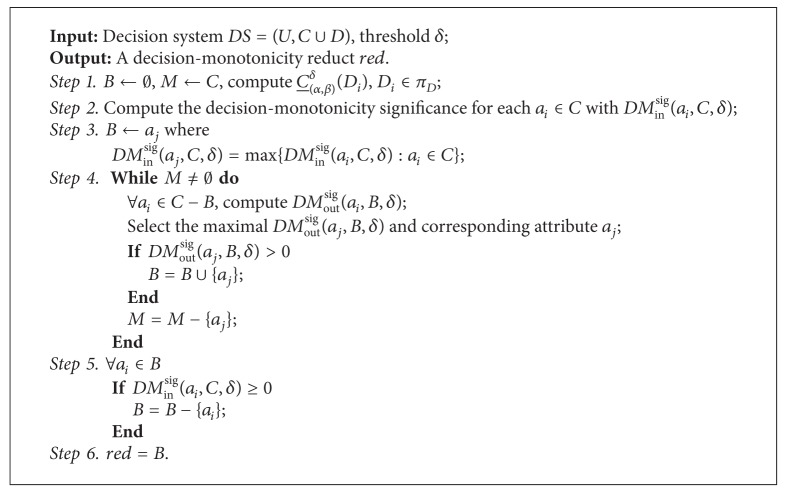
Heuristic algorithm for attribute reduction based on decision-monotonicity criterion.

**Algorithm 2:**
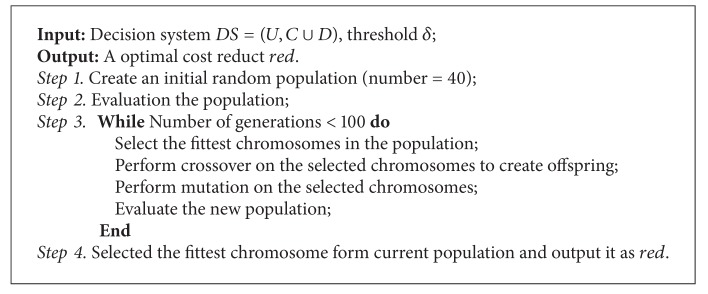
Genetic algorithm for attribute reduction based on cost minimum criterion.

**Table 1:** Data sets description.

ID	Data sets	Samples	Features	Decision classes
1	Annealing	798	38	5
2	Dermatology	366	34	6
3	Soybean	307	35	4
4	Zoo	101	17	7

**Table 2:** The decision rules between raw data and decision-monotonicity criterion based reducts (Annealing).

*δ*	(*P*) rules		(*B*) rules		(*N*) rules	
	Raw	Reduct	Raw	Reduct	Raw	Reduct
0.1	319.2 ± 412.1	**650.4 ± 311.2**	558.6 ± 385.5	**221.4 ± 356.5**	3112 ± 252.3	**3118 ± 233.4**
0.2	399 ± 420.6	**650.4 ± 311.2**	1117 ± 1077	**590.4 ± 582.1**	2473 ± 878.2	**2749 ± 516.0**
0.3	399 ± 420.6	**576.6 ± 356.5**	638.4 ± 733.3	**442.8 ± 622.3**	2952 ± 757.0	**2971 ± 498.1**
0.4	638.4 ± 336.5	**724.2 ± 336.5**	159.6 ± 336.5	**73.80 ± 233.4**	3192 ± 0.000	3192 ± 0.000
0.5	239.4 ± 385.5	**429.0 ± 388.9**	638.4 ± 504.7	**442.8 ± 516.0**	3112 ± 252.3	**3118 ± 233.4**
0.6	319.2 ± 412.1	**583.4 ± 346.6 **	798.0 ± 995.3	**503.6 ± 947.5**	2873 ± 770.9	**2903 ± 686.7**
0.7	363.5 ± 384.7	**523.7 ± 310.4**	611.3 ± 519.9	**438.2 ± 480.2**	3015 ± 335.2	**3028 ± 307.4**
0.8	379.3 ± 312.8	**648.9 ± 221.6**	729.8 ± 592.7	**437.9 ± 638.3**	2881 ± 417.8	**2903 ± 519.3**
0.9	713.2 ± 34.81	**727.5 ± 52.72 **	161.1 ± 54.24	**119.4 ± 96.12 **	3116 ± 39.46	**3143 ± 50.72**
1.0	798 ± 0	798 ± 0	0 ± 0	0 ± 0	**3192 ± 0 **	3118 ± 0

Mean values	456.8 ± 311.9	**631.2 ± 253.2**	541.2 ± 519.9	**334.4 ± 470.6**	2992 ± 370.4	**3024 ± 327.8**

**Table 3:** The decision rules between raw data and decision-monotonicity criterion based reducts (Dermatology).

*δ*	(*P*) rules		(*B*) rules		(*N*) rules	
	Raw	Reduct	Raw	Reduct	Raw	Reduct
0.1	0 ± 0	**1.100 ± 3.478**	**1134 ± 952.1**	1154 ± 848.9	**1061 ± 952.1**	1041 ± 847.3
0.2	0 ± 0	**0.500 ± 1.269**	**512.4 ± 602.6**	534.7 ± 583.3	**1683 ± 602.6**	1661 ± 582.9
0.3	0 ± 0	**0.200 ± 0.426**	512.3 ± 715.5	**448.8 ± 650.2**	1683 ± 715.5	**1747 ± 650.1**
0.4	0 ± 0	0.000 ± 0.000	878.2 ± 915.9	**860.4 ± 822.4**	1317 ± 915.9	**1335 ± 822.4**
0.5	0 ± 0	**0.4000 ± 0.699**	534.0 ± 703.5	**505.9 ± 643.8**	1662 ± 703.5	**1689 ± 643.5**
0.6	0 ± 0	**2.000 ± 0.943**	688.5 ± 754.3	**671.3 ± 779.7**	1507 ± 754.3	**1522 ± 779.3**
0.7	29.20 ± 5.473	**33.20 ± 12.35**	539.0 ± 522.5	**536.0 ± 525.2**	1627 ± 522.5	1627 ± 521.6
0.8	139.9 ± 5.953	139.9 ± 5.953	458.6 ± 314.6	458.6 ± 314.6	1597 ± 313.8	1597 ± 313.8
0.9	212.9 ± 9.036	219.9 ± 9.036	292.4 ± 124.3	292.4 ± 124.3	1691 ± 117.7	1691 ± 117.7
1.0	328.2 ± 1.932	328.2 ± 1.932	66.8 ± 24.14	66.8 ± 24.14	1801 ± 23.73	1801 ± 23.73

Mean values	71.02 ± 2.239	**71.84 ± 3.608**	561.7 ± 562.9	**552.8 ± 531.6**	1563 ± 562.2	**1571 ± 530.3**

**Table 4:** The decision rules between raw data and decision-monotonicity criterion based reducts (Soybean).

*δ*	(*P*) rules		(*B*) rules		(*N*) rules	
	Raw	Reduct	Raw	Reduct	Raw	Reduct
0.1	244.3 ± 128.7	**295.7 ± 20.52**	94.4 ± 149.0	**52.1 ± 98.76**	**889.3 ± 96.77**	880.2 ± 95.80
0.2	280.4 ± 43.59	**285.1 ± 30.39**	**85.2 ± 101.5**	93.2 ± 96.18	**862.4 ± 97.91**	849.7 ± 98.14
0.3	277.9 ± 8.212	**282.3 ± 14.11**	76.3 ± 73.26	**69.3 ± 78.64**	873.8 ± 67.97	**876.4 ± 69.86**
0.4	269.2 ± 2.821	**272.6 ± 12.33**	63.8 ± 19.17	**59.0 ± 27.68**	**895.0 ± 17.98**	825.9 ± 19.50
0.5	275.2 ± 5.827	**289.2 ± 15.64**	**81.1 ± 67.07**	112.9 ± 153.8	**871.7 ± 67.63**	825.9 ± 155.0
0.6	304.7 ± 2.312	**305.5 ± 1.581**	9.90 ± 14.65	**9.30 ± 14.96 **	**913.4 ± 15.04**	913.2 ± 14.98
0.7	302.9 ± 3.755	**305.7 ± 1.059**	5.70 ± 4.423	**2.80 ± 2.573**	919.4 ± 2.413	**919.5 ± 1.958**
0.8	298.2 ± 1.932	**304.8 ± 4.638**	17.5 ± 4.836	**5.70 ± 9.956 **	912.3 ± 3.713	917.5 ± 5.797
0.9	307 ± 0	307 ± 0	0 ± 0	0 ± 0	921 ± 0	921 ± 0
1.0	307 ± 0	307 ± 0	0 ± 0	0 ± 0	921 ± 0	921 ± 0

Mean values	286.6 ± 19.72	**295.5 ± 10.03**	43.39 ± 43.40	**40.43 ± 48.26**	**897.9 ± 36.92**	892.1 ± 46.11

**Table 5:** The decision rules between raw data and decision-monotonicity criterion based reducts (Zoo).

*δ*	(*P*) rules		(*B*) rules		(*N*) rules	
	Raw	Reduct	Raw	Reduct	Raw	Reduct
0.1	0 ± 0	**43 ± 0**	141.4 ± 70.62	**69.60 ± 59.90**	565.6 ± 70.72	**594.4 ± 59.90**
0.2	0 ± 0	**43 ± 0**	153 ± 55.23	**116 ± 66.97**	554 ± 55.23	548 ± 66.97
0.3	0 ± 0	**43 ± 0**	210.7 ± 197.1	**131 ± 127.1**	496.3 ± 197.1	**532.1 ± 127.1**
0.4	0 ± 0	**43 ± 0**	246.3 ± 216.9	**163.7 ± 158.5**	460.7 ± 216.9	**500.3 ± 158.5**
0.5	1.9 ± 3.143	**2.6 ± 3.406**	178.6 ± 173.9	**167.8 ± 126.4**	526.5 ± 174.1	**536.6 ± 124.3**
0.6	36.5 ± 3.689	36.5 ± 3.689	102.4 ± 68.08	102.4 ± 68.08	568.1 ± 69.54	568.1 ± 69.54
0.7	66.2 ± 8.377	**67.5 ± 8.657**	66.6 ± 39.14	**62.60 ± 43.91**	574.2 ± 38.29	**576.9 ± 41.53**
0.8	78.7 ± 8.795	78.7 ± 8.795	46.3 ± 20.95	46.30 ± 20.95	582 ± 15.24	582 ± 15.24
0.9	95.0 ± 0	95.0 ± 0	11.40 ± 0.9661	11.40 ± 0.9661	600.6 ± 0.9661	600.6 ± 0.9661
1.0	101 ± 0	101 ± 0	0 ± 0	0 ± 0	606 ± 0	606 ± 0

Mean values	37.93 ± 2.40	**55.33 ± 2.45**	115.7 ± 84.28	**87.17 ± 67.28**	553.4 ± 83.80	**564.5 ± 66.39**

**Table 6:** The comparison between decision-monotonicity criterion based reducts and cost based reducts (Annealing).

*δ*	Approximation qualities		Run times (s)	
	Algorithm 1	Algorithm 2	Algorithm 1	Algorithm 2
0.1	**0.8150 ± 0.3899**	0.4000 ± 0.5164	**12.43 ± 0.3997**	311.3 ± 25.18
0.2	**0.8150 ± 0.3899**	0.6401 ± 0.3762	**12.11 ± 0.0070**	261.8 ± 63.63
0.3	**0.7226 ± 0.4467**	0.6855 ± 0.2790	**12.12 ± 0.0143**	186.5 ± 26.66
0.4	**0.9075 ± 0.2925**	0.8397 ± 0.1122	**12.12 ± 0.0190**	205.6 ± 22.68
0.5	0.5376 ± 0.4874	**0.7153 ± 0.1350**	**12.12 ± 0.0076**	223.3 ± 29.21
0.6	0.7311 ± 0.4343	**0.8429 ± 0.0509**	**36.03 ± 39.38**	200.2 ± 8.255
0.7	0.6563 ± 0.3890	**0.9244 ± 0.0368**	**43.91 ± 40.84**	209.6 ± 40.87
0.8	0.8132 ± 0.2777	**0.9754 ± 0.0070**	**50.46 ± 42.55**	270.1 ± 67.62
0.9	0.9117 ± 0.0661	**0.9984 ± 0.0021**	**53.12 ± 36.45**	359.5 ± 106.0
1.0	1.0000 ± 0.0000	1.0000 ± 0.0000	**25.94 ± 32.02**	389.1 ± 129.9

Mean values	0.7910 ± 0.3174	**0.8022 ± 0.1516**	**27.04 ± 19.17**	261.7 ± 52.00

**Table 7:** The comparison between decision-monotonicity criterion based reducts and cost based reducts (Dermatology).

*δ*	Approximation qualities		Run times (s)	
	Algorithm 1	Algorithm 2	Algorithm 1	Algorithm 2
0.1	0.0030 ± 0.0095	**0.0913 ± 0.1191**	**2.524 ± 0.9840**	60.14 ± 12.11
0.2	0.0014 ± 0.0035	**0.1795 ± 0.0996**	**3.531 ± 2.9403**	47.88 ± 14.01
0.3	0.0005 ± 0.0012	**0.2197 ± 0.0345**	**4.444 ± 4.6011**	42.23 ± 3.884
0.4	0.0000 ± 0.0000	**0.2128 ± 0.0283**	**2.183 ± 0.1003**	43.43 ± 3.094
0.5	0.0011 ± 0.0019	**0.2014 ± 0.0166**	**6.589 ± 7.1960**	42.18 ± 3.991
0.6	0.0055 ± 0.0026	**0.3954 ± 0.1061**	**18.14 ± 0.2819**	46.62 ± 6.518
0.7	0.0907 ± 0.0338	**0.5462 ± 0.0488**	**17.71 ± 0.1935**	44.46 ± 5.502
0.8	0.3822 ± 0.0163	**0.5402 ± 0.0680**	**16.13 ± 0.7679**	49.32 ± 6.926
0.9	0.5817 ± 0.0247	**0.7533 ± 0.0358**	**13.91 ± 0.1070**	51.12 ± 10.22
1.0	0.8967 ± 0.0053	**0.8975 ± 0.0053**	**13.36 ± 0.0263**	58.57 ± 2.361

Mean values	0.1963 ± 0.0099	**0.4037 ± 0.0562**	**9.853 ± 1.719**	48.80 ± 6.863

**Table 8:** The comparison between decision-monotonicity criterion based reducts and cost based reducts (Soybean).

*δ*	Approximation qualities		Run times (s)	
	Algorithm 1	Algorithm 2	Algorithm 1	Algorithm 2
0.1	**0.9632 ± 0.0669**	0.9013 ± 0.0584	**6.976 ± 3.1845**	22.13 ± 4.443
0.2	0.9287 ± 0.0990	**0.9459 ± 0.0691**	**7.672 ± 2.3621**	23.36 ± 3.463
0.3	0.9195 ± 0.0460	**0.9492 ± 0.0578**	**7.638 ± 1.7622**	27.05 ± 6.543
0.4	0.8879 ± 0.0402	**0.9866 ± 0.0129**	**7.247 ± 2.2257**	28.93 ± 5.668
0.5	0.9420 ± 0.0510	**0.9948 ± 0.0054**	**2.217 ± 0.9900**	31.09 ± 8.096
0.6	**0.9951 ± 0.0052**	0.9896 ± 0.0103	**7.869 ± 0.1669**	33.91 ± 9.279
0.7	0.9958 ± 0.0035	**0.9964 ± 0.0047**	**6.398 ± 2.7240**	41.46 ± 6.722
0.8	0.9928 ± 0.0151	**1.0000 ± 0.0000**	**2.738 ± 2.6772**	37.79 ± 8.939
0.9	1.0000 ± 0.0000	1.0000 ± 0.0000	**7.529 ± 0.2508**	32.97 ± 9.095
1.0	1.0000 ± 0.0000	1.0000 ± 0.0000	**7.299 ± 0.2905**	35.63 ± 12.27

Mean values	0.9625 ± 0.0327	**0.9764 ± 0.0218**	**6.3586 ± 1.1663**	31.435 ± 7.453

**Table 9:** The comparison between decision-monotonicity criterion based reducts and cost based reducts (Zoo).

*δ*	Approximation qualities		Run times (s)	
	Algorithm 1	Algorithm 2	Algorithm 1	Algorithm 2
0.1	**0.4257 ± 0.0000**	0.2644 ± 0.2690	**0.0938 ± 0.0163**	4.2872 ± 0.4856
0.2	**0.4257 ± 0.0000**	0.2911 ± 0.3063	**0.0915 ± 0.0053**	4.4342 ± 0.1583
0.3	**0.4257 ± 0.0000**	0.3762 ± 0.2777	**0.0961 ± 0.0032**	4.7973 ± 0.4250
0.4	**0.4257 ± 0.0000**	0.3257 ± 0.2638	**0.0877 ± 0.0065**	4.4749 ± 0.4790
0.5	0.0257 ± 0.0337	**0.3277 ± 0.3271**	**0.2077 ± 0.1580**	4.1559 ± 0.3482
0.6	0.3614 ± 0.0365	**0.7129 ± 0.0417**	**0.3875 ± 0.0067**	4.4908 ± 0.5544
0.7	0.6683 ± 0.0857	**0.8554 ± 0.0896**	**0.3477 ± 0.0757**	5.2780 ± 1.1886
0.8	0.7792 ± 0.0871	**0.9564 ± 0.0344 **	**0.3747 ± 0.0244**	6.6658 ± 1.6919
0.9	0.9406 ± 0.0000	**1.0000 ± 0.0000**	**0.3857 ± 0.0048**	6.4638 ± 1.4479
1.0	1.0000 ± 0.0000	1.0000 ± 0.0000	**0.3799 ± 0.0143**	6.5578 ± 1.8033

Mean values	0.5478 ± 0.0243	**0.6110 ± 0.1610**	**0.2452 ± 0.0315**	5.1806 ± 0.8582
